# Crystal structure of the monoclinic phase (phase IV) of bis­(tetra­methyl­ammonium) tetra­chlorido­cuprate(II)

**DOI:** 10.1107/S2056989017002146

**Published:** 2017-02-14

**Authors:** Gorgui Awa Seck, Libasse Diop, Allen G. Oliver

**Affiliations:** a Laboratoire de Chimie Minérale et Analytique, Département de Chimie, Faculté des Sciences et Techniques, Université Cheikh Anta Diop, Dakar, Senegal; b Department of Chemistry and Biochemistry, University of Notre Dame, IN 46557-5670, USA

**Keywords:** crystal structure, tetra­hedral tetra­chlorido­cuprate ion, tetra­methyl­ammonium ion

## Abstract

The crystal structure of the low-temperature monoclinic phase of the title compound, [(CH_3_)_4_N]_2_[CuCl_4_], was determined at 120 K. The structure of the room-temperature phase has been determined in the ortho­rhom­bic space group *Pmcm* [Morosin & Lingafelter (1961 ▸). *J. Phys. Chem.* 50–51; Clay *et al.* (1975 ▸). *Acta Cryst.* B**31** 289–290]. The asymmetric unit consists of one discrete tetra­chlorido­cuprate anion with a distorted tetra­hedral geometry and two tetra­methyl­ammonium cations. In the crystal, the cations and the anions are linked *via* weak C—H⋯Cl hydrogen bonds.

## Abstract 2

The crystal structure of the low-temperature monoclinic phase of (Me_4_N)_2_[CuCl_4_] was determined at 120 K. The asymmetric unit consists of a discrete tetra­hedral [CuCl_4_]^2−^ anion and two crystallographically independent tetra­methyl­ammonium cations.

## Chemical context   

The title compound undergoes successive phase transitions at 297, 291 and 263 K (Sugiyama *et al.*, 1980 ▸). The room temperature phase (phase I) crystallizes in the ortho­rhom­bic space group *Pmcm* with Z = 4 (Morosin & Lingafelter, 1961 ▸; Clay *et al.*, 1975 ▸). Three low-temperature phases, named phases II, III and IV in the order of decreasing temperature, show incommensurate, ferroelastic commensurate monoclinic and monoclinic structures, respectively (Sugiyama *et al.*, 1980 ▸; Gesi & Iizumi, 1980 ▸). We allowed [(CH_3_)_4_N]Cl, CuCl_2_ and thio­acetamide to react in ethanol. The expected mixed ligand complex was not crystallized but instead the title compound was obtained accidentally. The crystal structure of phase IV of the title compound was determined at 120 K and is reported herein.
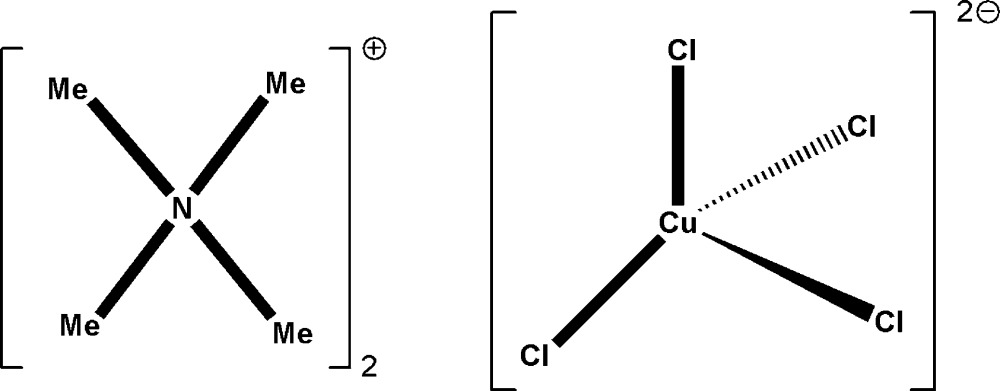


## Structural commentary   

**Figure 1:**
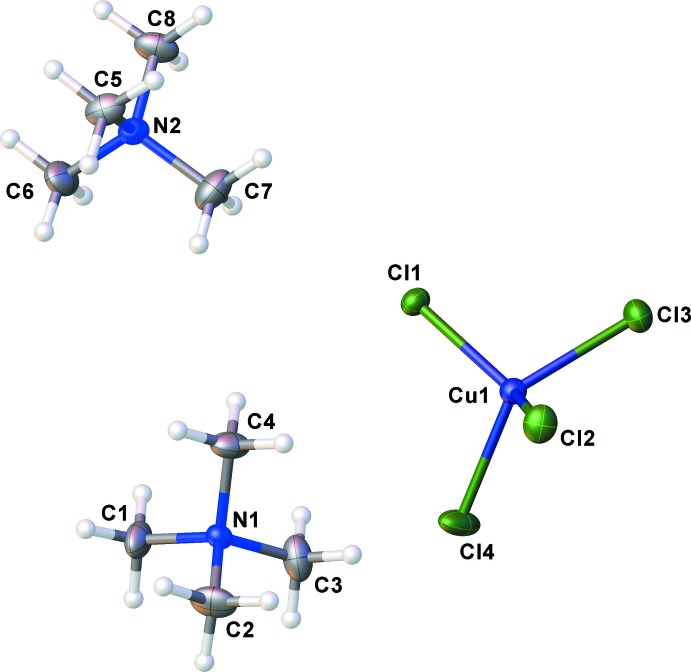
The asymmetric unit of the title compound showing the atom-labeling scheme. Displacement ellipsoids are drawn at the 50% probability level and H atoms are depicted as spheres of an arbitrary radius.

The asymmetric unit of the title compound consists of a discrete [CuCl_4_]^2−^ anion and two crystallographically tetra­methyl­ammonium cations (Fig. 1 ▸). In the anion, the four Cl atoms are inequivalent with Cu—Cl distances ranging from 2.2313 (15) to 2.2538 (16) Å. The Cl—Cu—Cl angles vary from 98.44 (7) to 133.69 (7)°, indicating a distorted tetra­hedral geometry around the Cu^II^ atom. Using Houser’s τ_4_ metric [τ_4_ = 360 − (α + β)/141], where α and β are the largest angles about the metal atom (Yang *et al.*, 2007 ▸), we obtain a value of 0.658 for phase IV and 0.792 for the ortho­rhom­bic phase I. This indicates a greater deviation from an ideal tetra­hedron in phase IV compared with phase I, tending towards a ‘see-saw’ geometry.

## Supra­molecular features   

**Figure 2:**
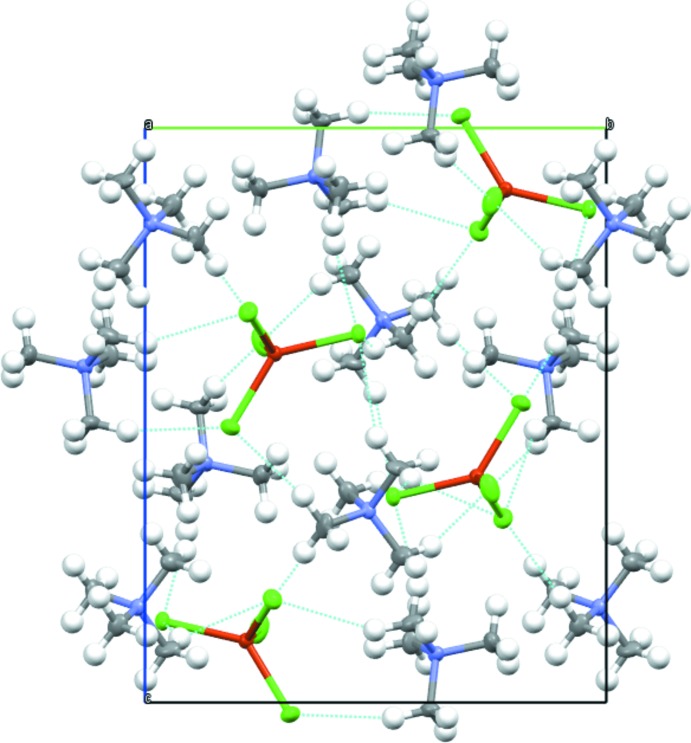
A packing diagram of the title compound, viewed along the *a* axis, showing the C—H⋯Cl hydrogen bonds (blue dashed lines).

In the crystal, the cations and the anions are linked *via* weak C—H⋯Cl hydrogen bonds (Table 1 ▸ and Fig. 2 ▸), forming a three-dimensional network.

## Database survey   

A substructure search for compounds that incorporate a tetra­methyl­ammonium ion and a copper tetra­chloride species reveals thirteen structures (CSD November 2016; Groom *et al.*, 2016 ▸). Of these, three are structures of (Me_4_N)_2_[CuCl_4_] with a discrete [CuCl_4_]^2−^ anion (Morosin & Lingafelter, 1961 ▸; Clay *et al.*, 1975 ▸; Hlel *et al.*, 2008 ▸).

## Synthesis and crystallization   

On mixing [(CH_3_)_4_N]Cl (0.465 g, 4.2 mmol) in ethanol (10 ml) with CuCl_2_·2H_2_O (0.365 g, 2.1 mmol) in ethanol (10 ml) and thio­acetamide (0.160 g, 2.1 mmol) in ethanol (10 ml), a clear solution is obtained. Slow evaporation at room temperature (301 K) yielded pale-green crystals of [(CH_3_)_4_N]_2_[CuCl_4_] suitable for X-ray determination.

## Refinement   

Crystal data, data collection and structure refinement details are summarized in Table 2 ▸. H atoms were included in idealized geometries and allowed to rotate to minimize their electron-density contribution with C—H = 0.98 Å and *U*_iso_(H) = 1.5*U*_eq_(C). The crystal used was found to be twinned through a 180° rotation about the reciprocal *a* axis with a twin component ratio of 0.76:0.24 (matrix: [1.000 −0.003 0.004 0.001 −1.000 −0.003 −0.093 0.005 −1.000]) . The diffraction data were integrated routinely applying this matrix and were scaled for absorption effects using *TWINABS* (Krause *et al.*, 2015 ▸). In the final model, incorporation of the twinned data did not significantly alter the model, thus the final model was refined using the majority component data.

## Supplementary Material

Crystal structure: contains datablock(s) I. DOI: 10.1107/S2056989017002146/is5469sup1.cif

Structure factors: contains datablock(s) I. DOI: 10.1107/S2056989017002146/is5469Isup2.hkl

CCDC reference: 1531866

Additional supporting information:  crystallographic information; 3D view; checkCIF report

## Figures and Tables

**Table 1:** Hydrogen-bond geometry (Å, °)

*D*—H⋯*A*	*D*—H	H⋯*A*	*D*⋯*A*	*D*—H⋯*A*
C1—H1*C*⋯Cl2^i^	0.98	2.69	3.585 (7)	153
C2—H2*A*⋯Cl1^ii^	0.98	2.79	3.675 (7)	151
C2—H2*A*⋯Cl3^ii^	0.98	2.80	3.555 (7)	134
C2—H2*B*⋯Cl1^i^	0.98	2.79	3.674 (7)	150
C3—H3*B*⋯Cl4	0.98	2.74	3.670 (7)	159
C4—H4*A*⋯Cl3^iii^	0.98	2.59	3.555 (7)	166
C5—H5*A*⋯Cl2^iv^	0.98	2.68	3.635 (7)	165
C5—H5*B*⋯Cl3^v^	0.98	2.81	3.587 (6)	137
C5—H5*C*⋯Cl4^i^	0.98	2.63	3.610 (6)	173
C8—H8*B*⋯Cl1^iii^	0.98	2.76	3.650 (6)	151
C8—H8*C*⋯Cl2^v^	0.98	2.82	3.763 (7)	162

Symmetry codes: (i) 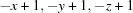; (ii) 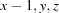; (iii) 
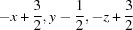
; (iv) 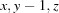; (v) 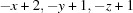.

**Table 2:** Experimental details

Crystal data	
Chemical formula	(C_4_H_12_N)_2_[CuCl_4_]
*M*_r_	353.63
Crystal system, space group	Monoclinic, *P*2_1_/*n*
Temperature (K)	120
*a*, *b*, *c* (Å)	8.9901 (5), 12.0059 (7), 14.9570 (9)
β (°)	91.719 (3)
*V* (Å^3^)	1613.65 (16)
*Z*	4
Radiation type	Mo *K*α
μ (mm^−1^)	1.99
Crystal size (mm)	0.15 × 0.12 × 0.11

Data collection	
Diffractometer	Bruker APEXII
Absorption correction	Multi-scan (*TWINABS*; Krause *et al.*, 2015 ▸)
*T*_min_, *T*_max_	0.659, 0.746
No. of measured, independent and observed [*I* > 2σ(*I*)] reflections	7842, 4019, 2862
*R*_int_	0.057
(sin θ/λ)_max_ (Å^−1^)	0.669

Refinement	
*R*[*F* ^2^ > 2σ(*F* ^2^)], *wR*(*F* ^2^), *S*	0.066, 0.141, 1.12
No. of reflections	4019
No. of parameters	144
H-atom treatment	H-atom parameters constrained
Δρ_max_, Δρ_min_ (e Å^−3^)	1.16, −1.03

Computer programs: *APEX3* and *SAINT* (Bruker, 2015 ▸), *SHELXT* (Sheldrick, 2015*a* ▸), *SHELXL2014* (Sheldrick, 2015*b* ▸), *XP* in *SHELXTL* (Sheldrick, 2008 ▸) and *publCIF* (Westrip, 2010 ▸).

## supplementary crystallographic information

### Crystal data

**Table:**

(C_4_H_12_N)_2_[CuCl_4_]	*F*(000) = 732
*M**_r_* = 353.63	*D*_x_ = 1.456 Mg m^−^^3^
Monoclinic, *P*2_1_/*n*	Mo *K*α radiation, λ = 0.71073 Å
*a* = 8.9901 (5) Å	Cell parameters from 6598 reflections
*b* = 12.0059 (7) Å	θ = 2.6–24.7°
*c* = 14.9570 (9) Å	µ = 1.99 mm^−^^1^
β = 91.719 (3)°	*T* = 120 K
*V* = 1613.65 (16) Å^3^	Block, pale green
*Z* = 4	0.15 × 0.12 × 0.11 mm

### Data collection

**Table:**

Bruker APEXII diffractometer	4019 independent reflections
Radiation source: fine-focus sealed tube	2862 reflections with *I* > 2σ(*I*)
Graphite monochromator	*R*_int_ = 0.057
Detector resolution: 8.33 pixels mm^-1^	θ_max_ = 28.4°, θ_min_ = 2.2°
combination of ω and φ–scans	*h* = −12→12
Absorption correction: multi-scan (*TWINABS*; Krause *et al.*, 2015)	*k* = −16→16
*T*_min_ = 0.659, *T*_max_ = 0.746	*l* = 0→19
7842 measured reflections	

### Refinement

**Table:**

Refinement on *F*^2^	Primary atom site location: real-space vector search
Least-squares matrix: full	Secondary atom site location: difference Fourier map
*R*[*F*^2^ > 2σ(*F*^2^)] = 0.066	Hydrogen site location: inferred from neighbouring sites
*wR*(*F*^2^) = 0.141	H-atom parameters constrained
*S* = 1.12	*w* = 1/[σ^2^(*F*_o_^2^) + 15.8242*P*] where *P* = (*F*_o_^2^ + 2*F*_c_^2^)/3
4019 reflections	(Δ/σ)_max_ < 0.001
144 parameters	Δρ_max_ = 1.16 e Å^−^^3^
0 restraints	Δρ_min_ = −1.03 e Å^−^^3^

### Special details

**Table:**

Geometry. All esds (except the esd in the dihedral angle between two l.s. planes) are estimated using the full covariance matrix. The cell esds are taken into account individually in the estimation of esds in distances, angles and torsion angles; correlations between esds in cell parameters are only used when they are defined by crystal symmetry. An approximate (isotropic) treatment of cell esds is used for estimating esds involving l.s. planes.

### Fractional atomic coordinates and isotropic or equivalent isotropic displacement parameters (Å^2^)

**Table:**

	*x*	*y*	*z*	*U*_iso_*/*U*_eq_	
Cu1	0.77037 (7)	0.71890 (6)	0.60734 (5)	0.01762 (17)	
Cl1	0.78370 (15)	0.53786 (11)	0.64164 (10)	0.0211 (3)	
Cl2	0.79189 (17)	0.81507 (13)	0.47896 (10)	0.0275 (3)	
Cl3	0.97801 (15)	0.77628 (13)	0.67958 (10)	0.0267 (3)	
Cl4	0.52836 (16)	0.74708 (14)	0.62755 (13)	0.0363 (4)	
N1	0.2548 (5)	0.4839 (4)	0.6659 (3)	0.0183 (10)	
C1	0.1601 (7)	0.3893 (5)	0.6925 (5)	0.0322 (15)	
H1A	0.0671	0.4177	0.7162	0.048*	
H1B	0.2128	0.3452	0.7386	0.048*	
H1C	0.1378	0.3422	0.6403	0.048*	
C2	0.1815 (7)	0.5491 (6)	0.5923 (4)	0.0328 (15)	
H2A	0.0840	0.5750	0.6113	0.049*	
H2B	0.1685	0.5017	0.5392	0.049*	
H2C	0.2436	0.6133	0.5779	0.049*	
C3	0.2801 (7)	0.5574 (6)	0.7446 (5)	0.0344 (16)	
H3A	0.1846	0.5868	0.7639	0.052*	
H3B	0.3451	0.6192	0.7284	0.052*	
H3C	0.3273	0.5146	0.7935	0.052*	
C4	0.4008 (6)	0.4411 (6)	0.6347 (5)	0.0296 (14)	
H4A	0.4516	0.4002	0.6834	0.044*	
H4B	0.4629	0.5038	0.6167	0.044*	
H4C	0.3836	0.3912	0.5836	0.044*	
N2	0.7505 (5)	0.1309 (4)	0.5840 (3)	0.0198 (10)	
C5	0.7809 (7)	0.1175 (6)	0.4871 (4)	0.0279 (14)	
H5A	0.7882	0.0381	0.4727	0.042*	
H5B	0.8748	0.1545	0.4738	0.042*	
H5C	0.6998	0.1511	0.4512	0.042*	
C6	0.6058 (7)	0.0788 (6)	0.6059 (5)	0.0334 (16)	
H6A	0.6112	−0.0018	0.5957	0.050*	
H6B	0.5264	0.1106	0.5675	0.050*	
H6C	0.5846	0.0931	0.6687	0.050*	
C7	0.7444 (8)	0.2520 (5)	0.6048 (5)	0.0347 (16)	
H7A	0.7225	0.2622	0.6681	0.052*	
H7B	0.6661	0.2872	0.5676	0.052*	
H7C	0.8405	0.2863	0.5925	0.052*	
C8	0.8722 (7)	0.0776 (6)	0.6381 (4)	0.0304 (15)	
H8A	0.8733	−0.0026	0.6260	0.046*	
H8B	0.8558	0.0901	0.7018	0.046*	
H8C	0.9678	0.1102	0.6222	0.046*	

### Atomic displacement parameters (Å^2^)

**Table:**

	*U*^11^	*U*^22^	*U*^33^	*U*^12^	*U*^13^	*U*^23^
Cu1	0.0169 (3)	0.0172 (3)	0.0188 (3)	0.0000 (3)	0.0018 (2)	0.0003 (3)
Cl1	0.0216 (7)	0.0157 (6)	0.0261 (7)	0.0009 (5)	0.0031 (5)	0.0006 (5)
Cl2	0.0355 (8)	0.0277 (8)	0.0195 (7)	−0.0040 (6)	0.0024 (6)	0.0043 (6)
Cl3	0.0227 (7)	0.0268 (8)	0.0304 (8)	−0.0034 (6)	−0.0019 (6)	−0.0028 (6)
Cl4	0.0169 (7)	0.0303 (8)	0.0621 (12)	0.0049 (6)	0.0097 (7)	0.0129 (8)
N1	0.014 (2)	0.018 (2)	0.022 (3)	0.0001 (18)	−0.0003 (18)	0.0018 (19)
C1	0.028 (3)	0.022 (3)	0.047 (4)	−0.010 (3)	0.012 (3)	−0.005 (3)
C2	0.030 (3)	0.048 (4)	0.020 (3)	0.013 (3)	−0.004 (3)	0.007 (3)
C3	0.038 (4)	0.037 (4)	0.028 (4)	−0.016 (3)	−0.001 (3)	−0.001 (3)
C4	0.020 (3)	0.030 (4)	0.038 (4)	0.006 (3)	0.008 (3)	0.007 (3)
N2	0.020 (2)	0.020 (2)	0.019 (3)	0.0022 (19)	0.0043 (19)	0.0018 (19)
C5	0.036 (4)	0.031 (3)	0.016 (3)	0.009 (3)	0.002 (3)	0.001 (3)
C6	0.021 (3)	0.031 (4)	0.048 (4)	−0.006 (3)	0.007 (3)	0.005 (3)
C7	0.051 (4)	0.022 (3)	0.032 (4)	0.001 (3)	0.009 (3)	−0.002 (3)
C8	0.025 (3)	0.043 (4)	0.023 (3)	0.009 (3)	0.001 (2)	0.010 (3)

### Geometric parameters (Å, º)

**Table:**

Cu1—Cl4	2.2313 (15)	C4—H4B	0.9800
Cu1—Cl1	2.2357 (15)	C4—H4C	0.9800
Cu1—Cl3	2.2374 (15)	N2—C8	1.486 (7)
Cu1—Cl2	2.2538 (16)	N2—C7	1.488 (8)
N1—C1	1.481 (7)	N2—C6	1.488 (7)
N1—C3	1.483 (8)	N2—C5	1.491 (7)
N1—C2	1.488 (7)	C5—H5A	0.9800
N1—C4	1.498 (7)	C5—H5B	0.9800
C1—H1A	0.9800	C5—H5C	0.9800
C1—H1B	0.9800	C6—H6A	0.9800
C1—H1C	0.9800	C6—H6B	0.9800
C2—H2A	0.9800	C6—H6C	0.9800
C2—H2B	0.9800	C7—H7A	0.9800
C2—H2C	0.9800	C7—H7B	0.9800
C3—H3A	0.9800	C7—H7C	0.9800
C3—H3B	0.9800	C8—H8A	0.9800
C3—H3C	0.9800	C8—H8B	0.9800
C4—H4A	0.9800	C8—H8C	0.9800
			
Cl4—Cu1—Cl1	99.33 (6)	N1—C4—H4C	109.5
Cl4—Cu1—Cl3	133.69 (7)	H4A—C4—H4C	109.5
Cl1—Cu1—Cl3	98.64 (6)	H4B—C4—H4C	109.5
Cl4—Cu1—Cl2	98.44 (7)	C8—N2—C7	109.7 (5)
Cl1—Cu1—Cl2	133.48 (6)	C8—N2—C6	109.5 (5)
Cl3—Cu1—Cl2	99.32 (6)	C7—N2—C6	109.1 (5)
C1—N1—C3	108.6 (5)	C8—N2—C5	109.2 (4)
C1—N1—C2	110.9 (5)	C7—N2—C5	108.5 (5)
C3—N1—C2	109.2 (5)	C6—N2—C5	110.8 (5)
C1—N1—C4	109.7 (5)	N2—C5—H5A	109.5
C3—N1—C4	109.6 (5)	N2—C5—H5B	109.5
C2—N1—C4	108.9 (5)	H5A—C5—H5B	109.5
N1—C1—H1A	109.5	N2—C5—H5C	109.5
N1—C1—H1B	109.5	H5A—C5—H5C	109.5
H1A—C1—H1B	109.5	H5B—C5—H5C	109.5
N1—C1—H1C	109.5	N2—C6—H6A	109.5
H1A—C1—H1C	109.5	N2—C6—H6B	109.5
H1B—C1—H1C	109.5	H6A—C6—H6B	109.5
N1—C2—H2A	109.5	N2—C6—H6C	109.5
N1—C2—H2B	109.5	H6A—C6—H6C	109.5
H2A—C2—H2B	109.5	H6B—C6—H6C	109.5
N1—C2—H2C	109.5	N2—C7—H7A	109.5
H2A—C2—H2C	109.5	N2—C7—H7B	109.5
H2B—C2—H2C	109.5	H7A—C7—H7B	109.5
N1—C3—H3A	109.5	N2—C7—H7C	109.5
N1—C3—H3B	109.5	H7A—C7—H7C	109.5
H3A—C3—H3B	109.5	H7B—C7—H7C	109.5
N1—C3—H3C	109.5	N2—C8—H8A	109.5
H3A—C3—H3C	109.5	N2—C8—H8B	109.5
H3B—C3—H3C	109.5	H8A—C8—H8B	109.5
N1—C4—H4A	109.5	N2—C8—H8C	109.5
N1—C4—H4B	109.5	H8A—C8—H8C	109.5
H4A—C4—H4B	109.5	H8B—C8—H8C	109.5

### Hydrogen-bond geometry (Å, º)

**Table:**

*D*—H···*A*	*D*—H	H···*A*	*D*···*A*	*D*—H···*A*
C1—H1*C*···Cl2^i^	0.98	2.69	3.585 (7)	153
C2—H2*A*···Cl1^ii^	0.98	2.79	3.675 (7)	151
C2—H2*A*···Cl3^ii^	0.98	2.80	3.555 (7)	134
C2—H2*B*···Cl1^i^	0.98	2.79	3.674 (7)	150
C3—H3*B*···Cl4	0.98	2.74	3.670 (7)	159
C4—H4*A*···Cl3^iii^	0.98	2.59	3.555 (7)	166
C5—H5*A*···Cl2^iv^	0.98	2.68	3.635 (7)	165
C5—H5*B*···Cl3^v^	0.98	2.81	3.587 (6)	137
C5—H5*C*···Cl4^i^	0.98	2.63	3.610 (6)	173
C8—H8*B*···Cl1^iii^	0.98	2.76	3.650 (6)	151
C8—H8*C*···Cl2^v^	0.98	2.82	3.763 (7)	162

Symmetry codes: (i) −*x*+1, −*y*+1, −*z*+1; (ii) *x*−1, *y*, *z*; (iii) −*x*+3/2, *y*−1/2, −*z*+3/2; (iv) *x*, *y*−1, *z*; (v) −*x*+2, −*y*+1, −*z*+1.
